# Manejo de las enfermedades pulmonares intersticiales difusas (EPID) asociadas a enfermedades autoinmunes, por el neumólogo en las diferentes unidades de EPID en España

**DOI:** 10.1016/j.opresp.2022.100160

**Published:** 2022-01-19

**Authors:** Orlando Acosta Fernández, Myriam Aburto Barrenetxea, Ana Belén Llanos González, María Jesús Rodríguez Nieto, María Molina Molina, Claudia Valenzuela

**Affiliations:** a Servicio de Neumología, Complejo Hospitalario Universitario de Canarias, La Laguna, Santa Cruz de Tenerife, España; b Servicio de Neumología. Hospital Universitario de Galdakao. Universidad del País Vasco, País Vasco, España; c Servicio de Neumología. Hospital Universitario Fundación Jiménez Díaz. CIBERES ISCIII, Madrid, España; d Servicio de Neumología. Hospital Universitario de Bellvitge, Barcelona, España; e Servicio de Neumología, Hospital Universitario La Princesa. Universidad Autónoma de Madrid, Madrid, España

**Keywords:** Enfermedad pulmonar intersticial difusa, Fibrosis pulmonar, Enfermedades autoinmunes sistémicas, Enfermedades reumáticas, Encuesta, Equipo multidisciplinar, Diffuse interstitial lung disease, Pulmonary fibrosis, Connective lung diseases, Rheumatic diseases, Survey, Multidisciplinary team

## Abstract

**Introducción:**

El objetivo del estudio fue conocer el manejo de los pacientes con enfermedad pulmonar intersticial difusa (EPID) asociada a enfermedades autoinmunes sistémicas (EAS) en las consultas de neumología especializadas en EPID en el territorio español.

**Metodología:**

El área de trabajo de EPID de la Sociedad Española de Neumología y Cirugía torácica (SEPAR) diseñó un cuestionario autocumplimentable de 25 preguntas, sobre aspectos relacionados con el diagnóstico y tratamiento de las EPID- EAS. Este se repartió entre los asistentes de la reunión de invierno del Área EPID y posteriormente vía e-mail a todos los integrantes del área de EPID de SEPAR. La participación fue anónima, voluntaria y sin contraprestación.

**Resultados:**

Participaron 74 neumólogos de 58 hospitales. El 77% disponía de una consulta especializada de EPID. Todas las unidades con acreditación SEPAR tenían un comité integrado por neumólogos y radiólogos y participación mayoritaria de patólogos y reumatólogos. En el 75% de los centros había una colaboración estrecha con reumatología para el manejo de las EPID-EAS. Un 85% consideró que la frecuencia de las EPID-EAS en las consultas está en aumento, siendo la más frecuente la EPID asociada a artritis reumatoide. El tratamiento de las EPID-EAS se decide consensuadamente entre neumología y reumatología en el 91,3% de los casos. El 67% de los neumólogos considera que los inmunosupresores y las terapias biológicas pueden enlentecer la progresión de las EPID-EAS. Un 51% utiliza antifibróticos en estas patologías.

**Conclusiones:**

La práctica totalidad de las unidades de EPID españolas acreditadas, por SEPAR ha establecido colaboraciones con reumatología para el adecuado manejo de los pacientes con EPID-EAS, habiéndose ido extendiendo esta práctica a unidades aun no acreditadas.

## Abstract (en)

**Introduction:**

The aim of the study was to know the management of patients with diffuse interstitial lung disease (ILD) associated with a systemic autoimmune diseases (SAD) in pulmonology outpatient clinics in Spain.

**Methodology:**

The ILD work area of the Spanish Society of Pulmonology and Thoracic Surgery (SEPAR) designed a self-completed questionnaire of 25 questions, on aspects related to the diagnosis and treatment of ILD-SAD. This was distributed among the attendees of the winter meeting of the ILD Area and later via e-mail to all the members of the ILD area of SEPAR. Participation was anonymous, voluntary and without consideration.

**Results:**

74 pulmonologists from 58 hospitals participated. 77% had a specialized ILD consultation. All Units with SEPAR accreditation had a committee made up of pulmonologists and radiologists and a majority participation of pathologists and rheumatologists. In 75% of the centers there was a close collaboration with Rheumatology for the management of ILD-SAD. 85% considered that the frequency of ILD-SAD consults is increasing, the most frequent being ILD associated with rheumatoid arthritis. The treatment of ILD-SAD is decided by consensus between pulmonologist and rheumatologist in 91.3% of the cases. 67% of pulmonologists consider that immunosuppressants and biological therapies can slow down the progression of ILD-SAD. 51% use antifibrotics therapies in these pathologies.

**Conclusions:**

Almost all of the accredited Spanish ILD Units by SEPAR have established collaborations with Rheumatology for the adequate management of patients with ILD-SAD, this practice having been extended to units not yet accredited.

## Introducción

La afectación parenquimatosa intersticial pulmonar, en sus diferentes formas, puede ser la primera, y en ocasiones única, manifestación de una enfermedad autoinmune sistémica (EAS). El reconocimiento de una base autoinmune en el estudio de una enfermedad pulmonar intersticial difusa (EPID) tiene relevancia en tanto que su enfoque terapéutico y pronóstico diferirá respecto a formas idiopáticas o a otras entidades clínicas con expresividad radiológica y/o histológica similar^1^.

La dificultad en la caracterización de estas entidades, especialmente cuando no presentan un cortejo reumático típico, supone un reto diagnóstico para el neumólogo. La ausencia de protocolos que generalicen la forma de proceder en el diagnóstico de las mismas hace presumir que su abordaje diagnóstico sea variable.

Las dificultades más señalables en su reconocimiento serían: una posible menor familiaridad y destreza de los neumólogos con la sintomatología y los signos sistémicos propios con los que pueden expresarse; la falta de estandarización en el panel de autoanticuerpos que deben ser analizados; las limitaciones existentes para una adecuada categorización, cuando la forma de expresión serológica y clínica es incompleta y la ausencia de fundamentos sobre la frecuencia con que deben repetirse los test serológicos a lo largo de la evolución de estos pacientes^2^.

Es por ello por lo que, teóricamente, parece deseable establecer sinergias de colaboración con los especialistas dedicados a las EAS, fundamentalmente reumatólogos e internistas subespecializados, con la intención de alcanzar un mejor conocimiento y aproximación diagnóstica a estas entidades y poder ofrecer la terapéutica más eficaz en cada caso^3^, ^4^.

Se estableció como hipótesis la consideración de que los equipos multidisciplinares de EPID que incorporan especialistas dedicados a las EAS, fundamentalmente reumatólogos e internistas subespecializados, mejoran la capacidad diagnóstica y terapéutica en estas enfermedades y, por tanto, el propósito de este estudio fue conocer el manejo que se sigue en las consultas especializadas de neumología y las unidades de EPID españolas, ante las EAS cuando coexisten con afectación intersticial pulmonar.

## Metodología

Varios neumólogos del comité ejecutivo del área de trabajo de EPID de la Sociedad Española de Neumología y Cirugía Torácica (SEPAR) diseñaron un cuestionario autocumplimentable que constaba de 25 preguntas, existiendo en algunas de ellas subpreguntas, con diferente formato y que incluían respuestas únicas o múltiples (Anexo I). Diez se refirieron a las características de la unidad de EPID de cada centro hospitalario y a la experiencia personal en la misma y cuatro a la prevalencia de las EPID asociadas a enfermedad autoinmune sistémica (EPID-EAS) en dichas consultas. El resto de ítems abordaban aspectos específicos sobre la estructura y organización para el diagnóstico, seguimiento y tratamiento de los pacientes con EPID-EAS.

La encuesta estaba dirigida a los neumólogos socios de SEPAR integrantes del área EPID. Inicialmente se repartió entre los asistentes a una mesa sobre EPID-EAS, durante la Reunión de invierno del Área EPID de la SEPAR (noviembre del 2019), difundiéndolo posteriormente vía correo electrónico al resto de miembros del área cumpliendo con las recomendaciones internacionales para encuestas distribuidas por internet. La participación fue anónima, voluntaria y sin contraprestación. Todos los participantes dieron su consentimiento informado de forma verbal o por e-mail a la entrega del cuestionario. La información obtenida en las encuestas no incluyó datos personales ni clínicos de pacientes. En todo momento se siguieron las normas de buena práctica y conducta en investigaciones realizadas con encuestas^5^. Para minimizar los sesgos, evitar respuestas duplicadas y conocer mejor la realidad del país, en aquellos hospitales con más de una encuesta rellenada y para aquellas preguntas referidas a la actividad de la unidad, se utilizaron los datos declarados por su coordinador y en su defecto del neumólogo con mayor tiempo de vinculación a la misma.

Se utilizaron métodos de estadística descriptiva usando frecuencias y porcentajes para cuantificar los resultados de las variables categóricas haciendo uso de la hoja de cálculo Excel de Microsoft Office 2010.

## Resultados

**Figura 1:**
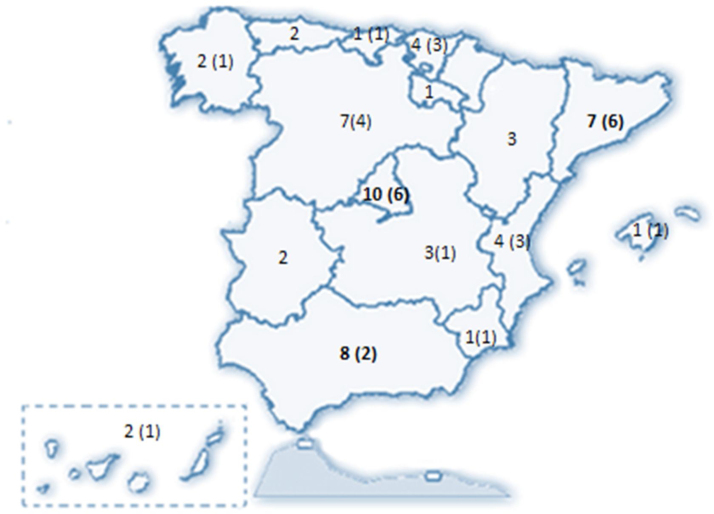
Servicios participantes en la encuesta, según Comunidades Autónomas y centros acreditados en EPID por SEPAR que participaron en la misma (en paréntesis). EPID: enfermedad pulmonar intersticial difusa; SEPAR: Sociedad Española de Neumología y Cirugía Torácica.

Se recibieron 77 encuestas, de las que se incluyeron 74 (dos fueron desestimadas al ser emitidas por facultativos no neumólogos y una tercera por no haberse identificado el centro de procedencia). Estos 74 neumólogos representaron a 58 servicios de neumología del país (Anexo II), distribuidos en 31 provincias de 15 de las 17 Comunidades Autónomas (CCAA) (fig. 1).

**Figura 2:**
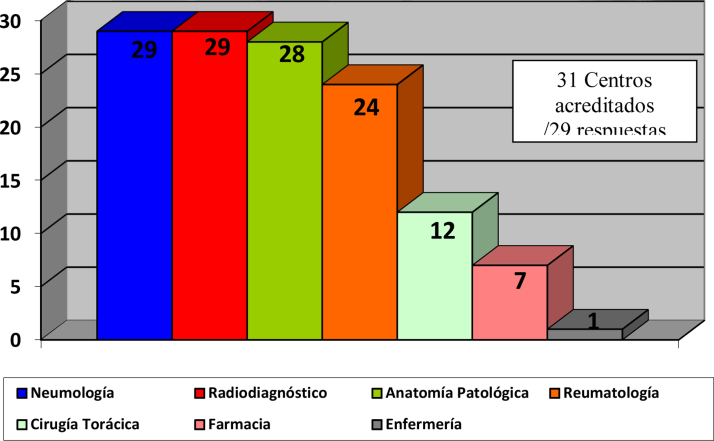
Especialidades presentes en las sesiones multidisciplinares de los centros acreditados por SEPAR que participaron en la encuesta. SEPAR: Sociedad Española de Neumología y Cirugía Torácica.

El 82,4% de los participantes trabajaba en los 45 (77,5%) hospitales con consulta monográfica de EPID. En la tabla 1 se recogen las características de los centros que respondieron a la encuesta. Los neumólogos con mayor experiencia en EPID trabajaban en centros con mayor nivel de acreditación SEPAR (p = 0,0188). Cuarenta y siete (81%) hospitales disponían de equipos multidisciplinares (EMD) para discutir el manejo diagnóstico de los pacientes. La frecuencia con que se reunía el EMD era mensual en 33 centros (70,2%), quincenal en 11 (23,4%) y semanal en tres (6,3%). En la fig. 2 se enumeran los especialistas que participan en los EMD. Los especialistas en EAS (generalmente reumatólogos) participan en el EMD de 40 centros (85,1%).

**Tabla 1:** Características de las consultas monográficas de EPID participantes en la encuesta

Tipo de consulta monográfica EPID	Acreditada SEPAR*			No acreditada SEPAR	Sin consulta monográfica	Total	Valor p
	Alta complejidad	Especializada	Básica				
n	8 (13,8)	15 (25,9)	8 (13,8)	14 (24,1)	13 (22,4)	58 (100)	
Características consulta EPID							
Experiencia del neumólogo en las consultas monográficas de EPID							0,0188
< 1año	0 (0)	0 (0)	0 (0)	2 (14,3)	np	2 (3,4)	
1-5años	0 (0)	1 (6,7)	5 (62,5)	7 (50)	np	13 (22,4)	
5-10 años	3 (37,5)	4 (26,7)	1 (12,5)	3 (23,4)	np	11 (19,0)	
> 10 años	5 (62,5)	10 (66,5)	2 (25)	2 (14,3)	np	19 (32,8)	
Número de pacientes valorados en las consultas monográficas de EPID a la semana							0,0025
0,0025	0 (0)	1 (6,7)	1 (12,5)	5 (35,7)	np	7 (12,1)	
0,0025	0 (0)	8 (53,3)	6 (75)	7 (70)	np	24 (41,4)	
0,0025	8 (100)	6 (40)	1 (12,5)	2 (14,3)	np	16 (27,6)	
Número de pacientes nuevos valorados en las consultas monográficas de EPID al mes							0,0092
1-5	0 (0)	0 (0)	2 (25)	2 (14,3)	np	4 (6,9)	
5-10	0 (0)	8 (53,3)	6 (75)	4 (28,6)	np	14 (24,1)	
> 10	8 (100)	7 (46,7)	0 (0)	8 (57,2)	np	23 (39,7)	
Herramientas diagnóstico EPID-EAS							
EMD para la discusión de los casos?							< 0,0001
	8 (100)	15 (100)	6 (75)	14 (100)	4 (30,8)	47 (81,0)	
Reumatólogo en el EMD							
	7 (87,5)	13 (86,6)	4 (50)	13 (92,8)	3 (23,1)	40 (69,0)	
Consultas conjuntas Neumología-Reumatología??							
	4 (50)	9 (60)	2 (25)	5 (35,7)	2 (15,4)	22 (37,9)	
Sesiones conjuntas Reumatología-Neumología							
	5 (62,5)	7 (46,6)	6 (75)	7 (42,8)	2 (15,4)	27 (46,5)	

Las variables numéricas expresan el número y porcentaje (entre paréntesis) de cada categoría. En la tabla solo se muestran aquellos resultados de p con diferencias estadísticamente significativas. Todos los resultados eran significativos a p < 0,05.

EPID: enfermedad pulmonar intersticial difusa; SEPAR: Sociedad Española de Neumología y Cirugía Torácica; EMD: equipo multidisciplinar; np: no procede; EPID-EAS: enfermedad pulmonar intersticial difusa asociada a enfermedad autoinmune sistémica; FAME: fármacos modificadores de la enfermedad reumática.

* Acreditación como unidad de EPID por la SEPAR.

? La frecuencia con que se reunían los EMD era mensual en 33 centros (70,2%), quincenal en 11 (23,4%) y semanal en tres (6,3%).

?? El 54% de los encuestados consideró que las consultas conjuntas, en las que ambos especialistas pasan a la vez, son necesarias. El 45% restante, afirmó que podían ser reemplazadas por sesiones clínicas compartidas.

Para el manejo y seguimiento de los pacientes con EPID-EAS, 22 (37,9%) de los centros participantes en la encuesta (tabla 1) organizaban consultas conjuntas entre los servicios de neumología y reumatología; mientras que en otros 27 (46,5%) hospitales se planificaban sesiones conjuntas, generalmente mensuales, entre ambas especialidades. El 54% de los encuestados consideró que las consultas conjuntas, en las que ambos especialistas pasan a la vez, son necesarias. El resto de los participantes (45%), las consideró de interés, aunque en su ausencia podían ser reemplazadas por sesiones clínicas compartidas.

La enfermería estaba integrada en 12 (20,6%) de las unidades de EPID participantes, habiéndose incorporado a las mismas, en más de la mitad de los casos, en los últimos tres años.

El 85% de los neumólogos consideró que la frecuencia de las EPID-EAS está incrementándose anualmente, mientras que un 3% opinó que se mantiene constante. El porcentaje de facultativos que no contestó esta pregunta fue un 12%.

De los 45 centros hospitalarios que aportaron información sobre el porcentaje aproximado que correspondía a las EPID-EAS en las consultas monográficas de EPID, 35 (77,7%) señalaron que estas representarían entre un 10-30% de las EPID, cinco centros (11,1%) estimaron que estas supondrían entre un 30-50% e igualmente cinco centros (11,1%) las cuantificaron como inferiores a un 10%.

Según las respuestas obtenidas, las EPID-EAS que los neumólogos valoran con más frecuencia en las unidades de EPID son las asociadas a artritis reumatoide (EPID-AR) seguidas, en este orden, por la EPID asociada a esclerosis sistémica (EPID-ES), las neumopatías intersticiales con características autoinmunes^6^ (denominadas habitualmente IPAF) y las miopatías inflamatorias.

En la tabla 2 se describen las pruebas que se realizan habitualmente al diagnóstico y durante el seguimiento de los pacientes con EPID-EAS. El 82% de los centros realiza el seguimiento funcional de los pacientes con periodicidad semestral, un 9% trimestral y el 9% restante de forma anual.

**Tabla 2:** Pruebas complementarias que se utilizan en la valoración inicial y en el seguimiento de los pacientes con EPID-EAS

Pruebas complemetarias	Valoración inicial	Durante el seguimiento	
		Siempre	Otras circunstancias
*PFR*?			
Espirometría	100%	100%	
DLCO	100%	100%	
Pletismografía	75%	57%	
*PM6M*	75%	74%	22%*
*TCAR torácico*	100%	19%	74%**
*Ecografía pulmonar*#	9%		

Las cifras muestran el % de neumólogos que utilizan las diferentes pruebas en la valoración inicial y durante el seguimiento de los pacientes con EPID-EAS, según los resultados de la encuesta.

PM6M: prueba de marcha de seis minutos; TCAR: tomografía computarizada de alta resolución.

? El 82% de los centros realiza el seguimiento funcional de los pacientes con periodicidad semestral, un 9% trimestral y el 9% restante de forma anual.

* Se utiliza solo en caso de presentar un test de difusión de monóxido de carbono (DLCO) < 50% y/o presentar una hipertensión pulmonar.

** Se realiza en caso de deterioro clínico funcional del paciente.

# Solo con fines de investigación.

El tratamiento de la EPID-EAS se decide de forma conjunta con los reumatólogos en el 91,3% de los casos. Solo en un 3% de los casos es el reumatólogo y en 1% el neumólogo quien decide de forma unilateral el tratamiento. El 67% de los neumólogos encuestados consideraron que los inmunosupresores (IS) o las terapias biológicas tienen el potencial de frenar la progresión de las EPID-EAS vs. un 22% que considera que no hay evidencia firme que contribuya a ello y el resto no se define.

La gran mayoría de los encuestados reconoció estar familiarizado con uno o más fármacos de uso común en el tratamiento de las enfermedades reumáticas, siendo la azatioprina o el micofenolato los de uso más frecuente (tabla 3).

**Tabla 3:** Uso de fármacos inmunomoduladores y antifibróticos para el tratamiento de las EPID-EAS según el tipo de consulta monográfica de EPID**

Tipo de consulta monográfica EPID	Acreditada SEPAR			No acreditada SEPAR	Sin consulta monográfica	Total	
	Alta complejidad	Especializada	Básica				
n	8 (13,8)	15 (25,9)	8 (13,8)	14 (24,1)	13 (22,4)	58 (100)	
FAMES tradicionales*	6 (75)	8 (53,3)	5 (62,5)	8 (57,2)	2 (15,4)	29 (50)	0,0249
Antiproliferativos (MM/AZA)	8 (100)	14 (93,3)	7 (87,5)	9 (64,3)	6 (46,2)	44 (75,9)	0,004
Anticalcineurínicos (C-A/TAC)	1 (12,5)	2 (13,4)	1 (12,5)	0	1 (7,7)	5 (8,6)	
Alquilantes (CYC)	7 (87,5)	7 (46,7)	6 (75)	5	3 (23,1)	28 (48,3)	0.0001
Biológicos	6 (75)	7 (46,7)	5 (62,5)	4 (28,6)	2 (15,4)	24 (41,4)	0,0071
Rituximab	1 (12,5)	5 (33,3)	2 (25)	2 (14,3)	1 (7,7)	11 (19)	
AntiTNF	1 (12,5)	-	-	1 (7,1)	-	2 (3,4)	
Abatacept	-	-	-	-	1 (7,7)	1 (1,7)	
Tocilizumab	-	-	-	-	-	-	
Varios	3(32,5)	2 (13,4)	3 (32,5)	1 (7,1)	-	9 (15,5)	
Antifibróticos	6 (75)	7 (46,7)	3 (32,5)	8 (57,2)	3 (23,1)	27 (46,5)	0,0066

Las variables numéricas expresan el número y porcentaje (entre paréntesis) de cada categoría. En la tabla solo se muestran aquellos resultados de p con diferencias estadísticamente significativas. Todos los resultados eran significativos a p < 0,05.

* FAMES tradicionales: metotrexato, leflunomida, sales de oro, hidroxicloroquina. MM: micofenolato de mofetilo; AZA: azatioprina; C-A: ciclosporina A; TAC: tacrolimus; CYC: ciclofosfamida; TNF: factor necrosis tumoral.

** El 67% de los neumólogos encuestados consideraron que los inmunosupresores o las terapias biológicas tienen el potencial de frenar la progresión de las EPID-EAS y el 86% de los encuestados conocen el potencial efecto de los fármacos antifibróticos en las EPID-EAS: un 51% los ha utilizado fuera de ficha técnica en estas patologías, fundamentalmente en EPID-ES, EPID-AR e IPAF (84, 65 y 36%, respectivamente).

El 86% de los encuestados conocen el potencial efecto de los fármacos antifibróticos en las EPID-EAS y un 51% los ha utilizado fuera de ficha técnica en estas patologías, fundamentalmente en EPID-ES, EPID-AR e IPAF (84, 65 y 36%, respectivamente).

## Discusión

Este es el primer trabajo que aporta una visión panorámica del manejo de las EPID-EAS por parte de los neumólogos españoles dedicados a las enfermedades intersticiales pulmonares. Los resultados de la encuesta permiten poner de manifiesto la relevancia que han tomado las EPID-EAS, que han pasado a ocupar el 10-30% de la patología de las consultas de EPID, circunstancia que ha favorecido la decidida incorporación del reumatólogo en las discusiones multidisciplinares que se realizan con finalidad diagnóstica en materia de EPID en nuestro país (85% de los centros encuestados). Además, un 75% de las unidades de EPID han desarrollado reuniones periódicas o consultas conjuntas específicas con el reumatólogo para controlar el tratamiento y seguimiento de estos pacientes.

Los resultados de la encuesta no solo ofrecen una amplia representación de centros hospitalarios distribuidos por casi todas las CCAA, sino también del 62% de las unidades acreditadas por SEPAR. El 82,5% de las respuestas fueron emitidas por neumólogos que trabajaban en consultas especializadas de EPID, teniendo un 55% de ellos, una experiencia superior a cinco años en el manejo de estas enfermedades. Una realidad positiva que hemos encontrado es que el 81% de los hospitales tienen equipos multidisciplinares con participación sistemática de neumólogos, radiólogos y patólogos, vs. el 56% de los especialistas en enfermedades intersticiales a nivel europeo^7^. La discusión multidisciplinar con la participación del neumólogo, radiólogo y patólogo mejoró la concordancia y confianza diagnóstica, en comparación con el diagnóstico inicial realizado por un experto individual^8^, especialmente para el diagnóstico de fibrosis pulmonar idiopática (FPI) y EPID-EAS^9^. En nuestro país, además, el reumatólogo forma parte del 85% de estos EMD, con un frecuencia muy superior a la descrita previamente en la literatura, donde solo siete de 29 equipos integraban a un reumatólogo (24,1%)^10^. Este hecho nos da idea de la relevancia y la preocupación de los neumólogos españoles en el correcto diagnóstico de las EPID-EAS en el marco del conjunto de la patología intersticial pulmonar^1^, ^6^, ^11^, ^12^. La importancia de incluir a un reumatólogo en el EMD ha quedado demostrado en algunos trabajos como el de Castelino et al.^13^, donde el 27% de los pacientes con EPID-EAS fueron diagnosticados inicialmente como FPI, y en un 38% la enfermedad reumatológica origen de la EPID estaba mal filiada, lo que originaba un cambio del tratamiento en el 80% de los pacientes con EPID-EAS y en un 27% de los pacientes con FPI.

En cuanto al porcentaje de casos que suponen las EPID-EAS, dentro de las consultas de EPID, la gran mayoría de neumólogos encuestados estimó que estas representarían actualmente entre un 10 y un 30% del total de pacientes con EPID de sus consultas, cifra que estaría en la línea con otros estudios epidemiológicos^14^, ^15^, que situarían a estas EPID entre el 14 y el 33% del espectro de las enfermedades intersticiales y ligeramente superior al encontrado por Xaubet et al. en el año 2001 en nuestro país^16^. En las consultas de neumología, las dos entidades más frecuentes de las EPID-EAS son, según los resultados de la encuesta, la EPID-AR y la EPID-ES, siendo ello coincidente con lo descrito en los estudios que han evaluado este aspecto, particularmente algunos registros de vida real^13^, ^17^, ^18^.

El rol de los EMD^12^, ^13^ se centra casi exclusivamente en el diagnóstico del paciente, si bien es verdad que el diagnóstico suele ser dinámico, y en ocasiones se reevalúan pacientes ante nuevos datos clínicos o radiológicos no acordes con el diagnóstico original. La función del EMD en el seguimiento del paciente es prácticamente inexistente, de ahí que para controlar la evolución de los pacientes con EPID-EAS se hayan debido crear nuevas plataformas, como las reuniones periódicas y sistematizadas entre neumólogos-reumatólogos o consultas conjuntas donde ambos especialistas evalúan al mismo tiempo al paciente con EPID-EAS, herramientas que están funcionando en el 75% de las unidades de EPID de los centros encuestados.

En la evaluación de los pacientes con EPID-EAS^19^, ^20^, las pruebas de función respiratoria (PFR) tienen un rol fundamental, tanto en la identificación de la enfermedad pulmonar, como en la evaluación de la gravedad de la EPID al diagnóstico y en la monitorización de la progresión durante el seguimiento, pero esta información siempre debe integrarse con todos los demás datos disponibles: clínicos y radiológicos^21^. En la presente encuesta se pone de manifiesto que, en la práctica clínica, la valoración de la función pulmonar se realiza principalmente mediante una espirometría y un estudio de la capacidad de difusión de monóxido de carbono (DLCO). Llama la atención la menor utilización de la pletismografía y la prueba de marcha de seis minutos (PM6M) en el seguimiento del curso de la enfermedad. Estos hallazgos pudieran estar explicados, en parte, por los requerimientos técnicos más complejos que se necesitan en estas dos últimas pruebas, pero también porque actualmente el concepto funcional de progresión de enfermedad está liderado por la capacidad vital forzada (FVC) y la DLCO^22^, ^23^, tanto en la propuesta específica de progresión de la EPID-EAS del grupo *Outcomes Measures in Rheumatoid Arthritis Clinical Trials* (OMERACT)^24^, como por el hecho de que la FVC ha pasado a considerarse como el objetivo apropiado para los estudios del desarrollo de fármacos en el tratamiento de la FPI y otras EPID^25^, ^26^. Sin embargo, debemos recalcar que una de las limitaciones de las PFR, es la experiencia necesaria para su interpretación, siendo recomendable no extraer conclusiones de las cifras aisladas que se obtengan en estas pruebas y sí integrarlas con otras variables, pudiendo ello plantear ciertas dificultades a facultativos no familiarizados. Entender el verdadero valor de un «cambio significativo» en las PFR y encajarlo en un contexto clínico-radiológico es fundamental para una correcta monitorización de la progresión de la enfermedad, por lo que se hace indispensable la evaluación multidisciplinar^21^.

Una de las ventajas de la PM6M, es que se trata de una técnica que mide de forma relativamente sencilla la capacidad funcional durante el ejercicio, siendo la distancia recorrida durante la misma la variable que se ha estimado más sólida como parámetro predictor evolutivo de estas enfermedades. Una reducción de la distancia recorrida de más de 50 m a lo largo de los últimos dos años se ha aceptado como expresión de progresión de la enfermedad con interés pronóstico^27^ y algunos autores introducen el comportamiento oximétrico durante la prueba y su relación con la distancia recorrida como estimador de supervivencia^28^. En la encuesta realizada, el 75% de los neumólogos encuestados solicitan regularmente la realización de una PM6M en la valoración inicial y durante el seguimiento de los pacientes con EPID-EAS.

Recientemente, se han definido nuevos criterios para evaluar la progresión de la enfermedad^29^, ^30^, ^31^, ^32^, entre los que se ha añadido el empeoramiento radiológico medido mediante la tomografía axial computarizada de alta resolución (TACAR), de ahí que un 20% de los neumólogos afirmen realizar una TACAR anual a sus pacientes, mientras que dos terceras partes de los neumólogos continúan aplicando las mismas recomendaciones que en el caso de pacientes con FPI, y que hacen uso del estudio radiológico mediante TACAR torácico exclusivamente cuando la situación clínica o funcional de sus pacientes sufre un empeoramiento^33^. Si bien hasta hace muy poco el seguimiento de los pacientes con EPID se basaba casi exclusivamente en la expresión clínica y en la evaluación funcional, hay una serie de circunstancias que podrían justificar la inclusión rutinaria de una prueba de imagen en el seguimiento de estos pacientes, como son la imposibilidad de obtener pruebas funcionales aceptables, estar ante enfermedades en fases incipientes en las que las variaciones funcionales podrían tener baja sensibilidad para interpretar cambios, disponer de una falsa normalidad en las cifras de FVC por la coexistencia de enfisema^34^, tener otros problemas que reducirían los volúmenes pulmonares como pueda ser la patología pleural o de caja torácica o tener un carga genética familiar aumentada de desarrollo de malignidad. Si bien no hay un posicionamiento oficial sobre esta práctica, se podría sentar la hipótesis de que una o varias de estas razones conducen a este grupo de neumólogos a practicar rutinariamente pruebas de imagen en el seguimiento de sus pacientes^35^.

En cuanto al manejo terapéutico, es relevante señalar que un 91,3% de los encuestados afirman consensuar el tratamiento de estos pacientes con el reumatólogo. La práctica totalidad de los neumólogos que se ocupan de las consultas de EPID tienen experiencia en el uso de fármacos inmunosupresores, siendo especialmente frecuente el manejo de azatioprina o micofenolato, y en menor proporción rituximab y ciclofosfamida. Llama también la atención que el 50% de los especialistas haya utilizado antifibróticos, en el tratamiento de pacientes con EPID-EAS. Aunque la evidencia de la eficacia y seguridad de la terapia con antifibróticos en estas patologías parece consolidarse, especialmente en los casos de EPID-ES y en EPID-EAS fibrosantes progresivas^26^, ^36^, debemos recordar que en el momento de enviar la encuesta estos fármacos no estaban aprobados por la agencia española del medicamento para estas indicaciones.

El presente estudio tiene como limitación principal que los datos presentados pueden no corresponder de forma rigurosa al manejo estándar o habitual de las EPID-EAS en la totalidad de los centros hospitalarios españoles, sobre todo en aquellos que no tengan una consulta específica de EPID. Sin embargo consideramos que sí reflejan el manejo de las EPID-EAS en las unidades acreditadas por SEPAR, puesto que hemos obtenido la respuestas del 80% de las unidades de alta complejidad, 65% de las unidades especializadas y 57% de las básicas. Estos datos, tampoco pueden ser generalizables a lo que ocurra en otros países o sistemas de salud, ya que la organización y recursos pueden diferir de forma sensible a los nuestros, ni podemos considerar las cifras emitidas por los encuestados como un estudio epidemiológico aunque se acerquen a las encontradas en estudios reales de este tipo.

Esta encuesta, en definitiva, da una visión del manejo en España de las EPID-EAS, sin que su propósito sea servir como guía de práctica clínica sobre el abordaje de estos pacientes. Consideramos que es un primer paso para identificar campos de mejora y poder establecer redes de trabajo con las que sentar las bases del adecuado manejo multidisciplinar de estos pacientes.

Las consultas y unidades especializadas de EPID forman parte de muchos de los servicios de neumología de la red pública española. La valoración multidisciplinar de los casos que se estudian y tratan en ellas es clave para su funcionamiento, con reuniones periódicas en las que han de participar neumólogos, radiólogos y patólogos, siendo de especial interés la incorporación de reumatólogos, dada la frecuencia con que se valoran EPID-EAS. Las EPID-EAS parecen ir en aumento en los últimos años en la actividad de la unidades de EPID, lo que pone en evidencia la necesidad de una colaboración estrecha entre neumólogos y reumatólogos para el correcto manejo de estas entidades.

## Consentimiento informado

Todos los participantes dieron su consentimiento informado de forma verbal o por e-mail a la entrega del cuestionario.

## Financiación

Este trabajo no ha recibido ningún tipo de financiación.

## Conflicto de intereses

Los autores declaran no tener ningún conflicto de intereses.
