# Supplementary material for: Management of Interstitial Lung Diseases (ILD) Associated with Autoimmune Diseases by the Pulmonologyst in the Differents ILD Units in Spain
Source: Open Respir Arch. 2022 Jan 19;4(1):100160. [Article in Spanish] doi: 10.1016/j.opresp.2022.100160 (PMC10369650; doi:10.1016/j.opresp.2022.100160)
Supplement: Supplementary file 1 [file mmc1.pdf]

ANEXO I. CUESTIONARIO SOBRE EL MANEJO DE LAS EPID DE BASE AUTOINMUNE

1. ¿Dispone tu Centro de una consulta monográfica de EPID?

a)Sí b) No

En caso afirmativo

-¿Cuántos pacientes suelen verse cada semana?

a) < 15 b) Entre 15 y 30 c) Más de 30

-¿ Cuántos pacientes nuevos pueden verse cada mes?

a) < 5 b) De 5 a 10 c) Más de 10

2- ¿Está tu centro/ Unidad de EPID acreditado por SEPAR? Señalar lo que proceda

a) Unidad básica

b) Unidad especializada

c) Unidad Multidisciplinar de alta complejidad

d) con grado de excelencia.

3. ¿Se realizan en tu centro, <u>de forma regular</u>, sesiones multidisciplinares en enfermedades intersticiales? a) Sí b) No

En caso afirmativo

3A. Con qué frecuencia se realizan

a) 1/ mes b) 1/15 días c) 1/ semana d Más de una semanal

3B. Señalar los facultativos que participan regularmente en las mismas

a) Neumólogo/a b) Radiólogo/a c) Patólogo/a d) Reumatólogo/a

e) Cirujano/a torácico f) Internista g) Farmacéutico h) Paliativista i) Otros

4. ¿Se realizan en tu centro, <u>de forma regular,</u> sesiones clínicas específicas con el Servicio de Reumatología para comentar casos con EPID de base autoinmune?

a) Sí b) No

En caso afirmativo

- Con qué frecuencia se realizan

a) 1/ mes b) 1/15 días c) 1/ semana

5. ¿Se realizan regularmente en tu servicio <u>consultas conjuntas</u> Reumatología/Neumología para la valoración específica de estos pacientes?

a) Sí b) No

En caso afirmativo

- Con qué frecuencia se llevan a cabo este tipo de consultas

a) 1/ mes b) 1/15 días c) 1/ semana d Más de una semanal

6. Consideras que las <u>consultas conjuntas</u> para la valoración y tratamiento de EPID de base autoinmune….

a) No son necesarias

b) Son necesarias

c) Son de interés pero pueden reemplazarse por las sesiones regladas y conjuntas entre reumatólogo- neumólogo

7. ¿Cuál es el papel del neumólogo con las EPID asociadas a enfermedad autoinmune, en tu propio centro? (elegir una o varias respuestas)

a) Realizar screening y establecer el diagnóstico de la EPID asociadas a EAS ya definida

b) Durante el estudio de una EPID con datos de autoinmunidad (IPAF), remitir al reumatólogo para confirmar el diagnóstico de la EAS y si no se confirma una EAS definida continua el neumólogo con el seguimiento de dicho paciente

c) Durante el estudio de una EPID con datos de autoinmunidad (IPAF), remitir al reumatólogo para confirmar el diagnóstico de la EAS y si aunque no se confirme una EAS definida el reumatólogo continua con el seguimiento de dicho paciente

d) Decidir y realizar el tratamiento de la EPID asociada a EAS sin participar el reumatólogo/internista.

e) El tratamiento de la EPID es decidido y realizado por el reumatólogo/ internista.

f) El tratamiento de la EPID se decide de forma conjunta entre el neumólogo y reumatólogo/internista

g) El neumólogo realiza el seguimiento, clínico-funcional y define gravedad y progresión de la EPID asociada a EAS

h) El reumatólogo/internista realiza el seguimiento, clínico-funcional y define gravedad y progresión de la EPID asociada a EAS

8. En tu consulta de EPID ¿qué porcentaje de pacientes que están en seguimiento corresponden a EPID asociadas a enfermedad autoinmune (conectivopatías)?

a) Menos del 10%
b) Del 10% al 25%
c) Del 30% al 50%
d) Más del 50%

9. En tu consulta de EPID ¿qué porcentaje de pacientes que están en seguimiento corresponden a pacientes clasificados como IPAF?

a) Menos del 10%
b) Del 10% al 20%
c) No tengo los datos

10. Según vuestra experiencia en la consulta de EPID los pacientes NUEVOS derivados desde Reumatología-Medicina Interna:

a) Está creciendo cada año.
b) No ha variado con los años.
c) Está disminuyendo.
d) No lo sé, no tengo datos

11. ¿Cuáles son las EPID de base autoinmune que ves más frecuentemente en la consulta? (numéralas en orden de importancia siendo 1 la más frecuente y 5 la menos frecuente)

a) Esclerosis sistémica
b) Artritis Reumatoide
c) Miopatías inflamatorias
d) IPAF
e) Otras

12. ¿Con qué frecuencia realizas habitualmente el control funcional respiratorio a los pacientes con EPID de base autoinmune que están en seguimiento en tu consulta?

a) Cada 3 meses
b) Cada 6 meses
c) Una vez al año en función de la severidad de la EPID

13. ¿Con qué frecuencia se realiza control radiológico con TACAR de tórax en las EPID de base autoinmune?

a) solo al diagnóstico
b) una vez al año
c) solo si hay empeoramiento clínico o funcional.
d) otras……………………………………………………………………..

14. ¿Tienes experiencia personal en ecografía torácica para pacientes con EPID o sospecha de EPID?

a) Sí b) No

En caso afirmativo, contestar lo que proceda

a) Suelo hacer un uso asistencial de ella para apoyar la sospecha de EPID
b) Suelo hacer un uso asistencial de ella para el seguimiento del paciente con EPID
c) Hago uso de ella solo con fines de investigación

15. ¿Realizas en el seguimiento de los pacientes con EPID de base autoinmune test de la marcha de 6 min?

a) si, a todas/casi todas
b) no, solo a las que presentan datos de hipertensión pulmonar en ecocardiografía
c) no, solo a las que presenta una DLCO por debajo del 50% y/o FVC < 50%
d) nunca/ no realizo test de marcha en el seguimiento

16. ¿Cuál/Cuáles son las Pruebas de función respiratoria que realizas para el estudio inicial – screening de las EPID asociada a EAS?

a) Espirometría
b) Espirometría y difusión
c) Espirometría , difusión y pletismografía
d) Espirometría, difusión y prueba de marcha de 6 min
e) Espirometría, difusión, pletismografía y prueba de marcha de 6 min

17. ¿Cuál/Cuáles son las Pruebas de función respiratoria que realizas para seguimiento de las EPID asociada a EAS?

a) Espirometría
b) Espirometría y difusión
c) Espirometría , difusión y pletismografía
d) Espirometría, difusión y prueba de marcha de 6 min
e) Espirometría, difusión, pletismografía y prueba de marcha de 6 min

18. ¿Utilizas habitualmente como tratamiento en tu práctica clínica los siguientes fármacos? Señalar los que proceda

a) Metotrexato
b) Sulfasalazina
c) Leflunomida
d) Micofenolato o ac micofenólico
e) Ciclofosfamida
f) Ciclosporina
g) Ninguno de ellos

19. ¿Prescribes en tu práctica clínica habitual los tratamientos inmunosupresores parenterales? Señalar los que procedan

a) Ciclofosfamida
b) Ciclosporina
c) Tacrolimus
d) Ninguno de ellos

20. ¿Prescribes en tu práctica clínica habitual los tratamientos biológicos? Señalar los que procedan

a) Inhibidores TNF-a (Infliximab, Adalimumab, Etanercept).
b) Moduladores de coestimulación selectiva (Abatacept)
c) Inhibidores selectivos de linfocitos B (Rituximab)
d) Inhibidores IL-6 (Tocilizumab)
e) Ninguno de ellos

21. Con la evidencia que existe hasta ahora de eficacia y seguridad de las terapias inmunosupresoras y biológicas en el tratamiento de las EPID asociadas a EAS, dirías que:

a) Frenan o enlentecen la progresión de la EPID con un perfil de seguridad favorable.

b) Frenan o enlentecen la progresión de la EPID con un perfil de seguridad a tener en cuenta debido a los potenciales efectos secundarios que presentan.

c) No mejoran la progresión de la EPID y los efectos adversos de los fármacos deben tenerse en cuenta.

d) No hay clara evidencia de que produzcan mejoría de la progresión de la EPID y además el perfil de seguridad de dichos fármacos debe tenerse en cuenta, debido a los potenciales efectos secundarios que presentan.

22. ¿Cuántos años de experiencia tiene en la utilización de los tratamientos antifibróticos existentes para el tratamiento de la FPI?

a) Menos de 1 año
b) Entre 1 y 3 años
c) más de 3 años
d) más de 5 años

23. En tu servicio/Unidad, ¿cuentas con una consulta de enfermería para el manejo de los potenciales efectos secundarios de los fármacos antifibróticos que se utilizan habitualmente para el tratamiento de la FPI?

a) Si b) No

Si la respuesta es afirmativa, cuánto tiempo lleva funcionando esta consulta:

a-Menos de 1 año

b-Entre 1 y 3 años

c- más de 3 años

d- más de 5 años

24. ¿Estás informado de la evidencia existente con fármacos antifibróticos para el tratamiento de las EPID asociadas a EAS?

a) Si b) No

25. ¿Tienes experiencia en tu servicio /unidad con la prescripción de antifibróticos como "uso compasivo" en una EPID asociada a enfermedad autoinmune?

a) Si b)No

Si la respuesta es afirmativa, en cual/cuáles:

a-Esclerosis sistémica

b-Artritis Reumatoide

c-Miopatías inflamatorias

d- IPAF

e-Otras

ANEXO II. CENTROS HOSPITALARIOS PARTICIPANTES

1. Clínica Cruz Blanca
2. Clínico Universitario Valladolid
3. Complejo Hospitalario Ourense
4. Complejo Universitario Albacete
5. Complejo Universitario León
6. Hospital General Universitario Valencia
7. Hospital de Gran Canaria Dr. Negrín
8. Hospital Universitario Basurto
9. Hospital 12 de Octubre
10. Hospital Arnau de Vilanova
11. Hospital Bellvitge
12. Hospital Ciudad de Jaen
13. Hospital Clínico San Carlos
14. Hospital Clínico Universitario Lozano Blesa
15. Hospital Clínico Valencia
16. Hospital de Valme
17. Hospital del Mar
18. Hospital El Bierzo
19. Hospital Fundación Jiménez Díaz
20. Hospital Germans Trias i Pujol
21. Hospital Gómez Ulla
22. Hospital Infanta Leonor
23. Hospital Infanta Sofia
24. Hospital Llerena
25. Hospital Lucus Augusti
26. Hospital Marqués de Valdecilla
27. Hospital Mataró
28. Hospital Miguel Servet
29. Hospital Móstoles
30. Hospital Ntra Sra de Sonsoles
31. Hospital Ntra Sra del Prado
32. Hospital Princesa
33. Hospital Puerta de Hierro
34. Hospital Punta de Europa
35. Hospital Ramón y Cajal
36. Hospital Regional Universitario de Málaga
37. Hospital Río Hortega
38. Hospital San Cecilio
39. Hospital San Pau
40. Hospital San Pedro
41. Hospital Sant Joan Despí Moisés Broggi
42. Hospital Santa Ana
43. Hospital Son Espasses
44. Hospital Universitario Galdakao-Usansolo
45. Hospital Universitario Salamanca
46. Hospital Universitario Alvarez-Buylla
47. Hospital Universitario Araba
48. Hospital Universitario Burgos
49. Hospital Universitario Cáceres
50. Hospital Universitario Cruces
51. Hospital Universitario de Canarias
52. Hospital Universitario San Agustín
53. Hospital Universitario Virgen de la Arrixaca
54. Hospital Virgen de la Salud
55. Hospital Virgen de las Nieves
56. Hospital Virgen del Rocío
57. Hospital General Universitario Alicante
58. Hospital Clinic
